# Decision tree accelerated CTU partition algorithm for intra prediction in versatile video coding

**DOI:** 10.1371/journal.pone.0258890

**Published:** 2021-11-08

**Authors:** Guowei Teng, Danqi Xiong, Ran Ma, Ping An

**Affiliations:** School of Communication and Information Engineering, Shanghai University, Shanghai, China; Nanjing University of Information Science and Technology, CHINA

## Abstract

Versatile video coding (VVC) achieves enormous improvement over the advanced high efficiency video coding (HEVC) standard due to the adoption of the quadtree with nested multi-type tree (QTMT) partition structure and other coding tools. However, the computational complexity increases dramatically as well. To tackle this problem, we propose a decision tree accelerated coding tree units (CTU) partition algorithm for intra prediction in VVC. Firstly, specially designated image features are extracted to characterize the coding unit (CU) complexity. Then, the trained decision tree is employed to predict the partition results. Finally, based on our newly designed intra prediction framework, the partition process is early terminated or redundant partition modes are screened out. The experimental results show that the proposed algorithm could achieve around 52% encoding time reduction for various test video sequences on average with only 1.75% Bjontegaard delta bit rate increase compared with the reference test model VTM9.0 of VVC.

## Introduction

With the advancements in multimedia technologies and video market, Ultra-High Definition (UHD) videos are becoming more and more popular due to their high resolution and extensive dynamic-range of luminance. The advanced High efficiency video coding (HEVC) standard [1] does not have sufficient compression ability to satisfy the rapid growth of data. In order to investigate the potential need for standardization of future video coding technology, the Moving Picture Experts Group (MPEG) and Video Coding Experts Group (VCEG) have established a collaborative Joint Video Exploration Team (JVET), which has published Call for Proposals (CfP) [2] for the next generation video coding standard–– H.266/VVC [3]. In the middle of 2020, the JVET has released the latest version video test model (VTM10.0) of H.266/VVC [4]. With the development of JVET, many new coding techniques have been studied and adopted, such as quad-tree plus binary tree (QTBT) block partition structure [5], intra sub-partitions (ISP) [6], multiple reference line (MRL) intra prediction [7], and adaptive multiple core transform [3], etc. It is reported that such tools significantly enhance the performance over HEVC at the cost of a sharp increase in complexity. According to the latest report by JVET, the intra coding complexity of VVC test software (VTM10.0) increases by 25 times compared with that of HEVC test software HM16.22 under All-Intra test configuration [4, 8]. Therefore, it is crucial to reduce coding complexity while maintaining coding performance, so as to achieve a balance between them.

**Fig 1:**
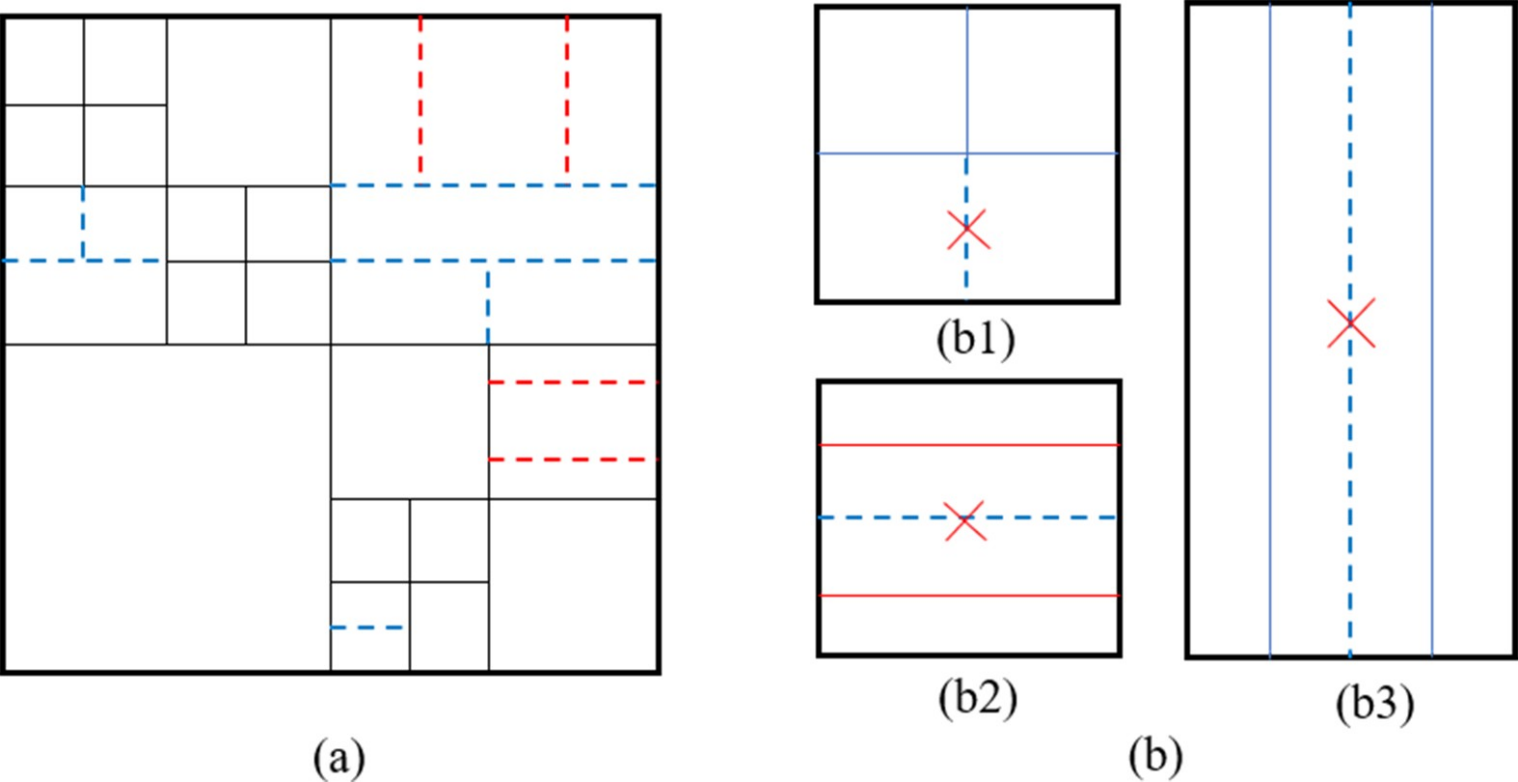
QTMT partition diagram: (a) the final partition of a CTU. (b) some restrictions for MT partition.

Similar to HEVC, VVC also uses block-based hybrid coding framework. Intra prediction, as an extremely important part, occupies a large proportion of coding time consumption. Different from HEVC, the quad-tree with nested multi-type tree (QTMT) structure is used in H.266/VVC to adapt the characteristics of various texture patterns. Since the new QTMT structure, the CU shape could be square or rectangular. There are four partition types in multi-type tree (MT) structures, including vertical binary tree partition (BV), horizontal binary tree splitting (BH), vertical ternary tree splitting (TV), and horizontal ternary tree splitting (TH). A brief illustration of QTMT structure is shown in Fig 1(A). The solid black lines represent quad-tree (QT) partition, and the colored dotted lines represent MT partition. Specifically, the red lines indicate TH/TV and the blue lines indicate BH/BV. There exist redundant CU splits that are forbidden in reality. Fig 1(B) shows several restriction examples. Case (b1) is the circumstance when a QT node is split by BH and its upper sub-CU is split by BV, the lower sub-CU cannot be split by BV anymore. Case (b2) is the circumstance when a QT node is split by TH, the second sub-part cannot be further split by BH because the final partition result is the same as that of two consecutive BH splitting. Case (b3) is an asymmetric version of case (b2). With the adoption of MT, the process of obtaining the best CTU structure is more complicated than that of HEVC.

Over the years, many academics have been worked on accelerating the intra prediction decision process in HEVC and its extensions, VVC. Although various fast algorithms have been invented for HEVC in previous work, they cannot be transplanted to VVC directly since the change of partition structure and the increase of the number of intra prediction modes. To address these problems, this paper further optimizes and improves the current VTM intra prediction framework, uses decision tree to predict the QT and MT partition or termination of the CU, and screens out candidate partition modes. In order to ensure the prediction accuracy of the decision tree, this paper also analyzes the selection of different features and finally chooses four types of features for off-line training of the decision tree. The confidence threshold of our trained two types of decision trees can be manually set according to specific needs. The experimental results show that our proposed algorithm achieves a good balance between rate-distortion (RD) performance and complexity reduction for various sequences, especially the UHD sequence.

The rest of this paper is organized as follows. The state-of-the-art fast intra-coding with optimized block partitioning methods are illustrated in Section II. Section III analyzes statistical distributions of CU depth and block size in intra coding mode. Then, the relationships between the complexities and CU partitions are investigated. Section IV presents our decision tree based partition mode prediction and early termination cascade decision framework for intra prediction in VVC, various image features are utilized for decision tree training including global texture complexity, local sub-CUs texture complexity, current CU context information, neighboring CU reference information. The experimental results and analysis are given in Section V, which could confirm our proposed algorithm theoretically and practically. Finally, the conclusions are drawn in Section VI.

### Related works

Over the years, plenty of researchers have devoted their efforts to the reduction of the computational complexity for intra prediction no matter based on the predecessor of H.266/VVC, H.265/HEVC, H.264/AVC, or coding standards proposed by other organizations. Due to the proposed time about VVC, there are still not too many related studies. On the contrary, as a predecessor and closest version to VVC, HEVC has lots of research results that are worth learning from on fast intra-coding with optimized block partitioning.

Specifically speaking, these approaches can be divided into five categories, including encoding information based, texture and edge complexity based, neighboring blocks based, RD cost based and machine learning based methods. Many approaches involve in some of the above categories, but not all of them.

### A. Encoding information based methods

The first category speeds up the block partitioning decision process by referring to the middle parameters in the encoding process, such as the coded block flag (CBF), sample adaptive offset (SAO) parameters, etc. For example, Gweon et al. [9] propose a CBF based early termination method by detecting zero CBFs to skip the remaining partitions of the current CU. In [10, 11], the energy of the prediction residual block is utilized to early terminate the CU recursive splitting process. In [12], Sangsoo effectively utilizes the side information such as SAO parameter values, PU sizes, CBF data, and MV sizes to minimize the process of CU split decision.

### B. Texture and edge complexity based methods

By utilizing the correlations between the texture and edge complexity of the video content and the block partitioning results, CU/PU split process can be accelerated greatly. Regularly, the area with detailed texture is more likely to be split into many small blocks to obtain better prediction, and on the contrary, the homogenous area is more inclined to no longer be divided. Besides, the direction and gradient of a block can reflect its texture complexity and edge information. Therefore, many related works have been done. Shen et al. [13] propose a fast CU size decision algorithm by measuring texture homogeneity and obtaining the appropriate thresholds with off-line training. For intra coding, Zhang et al. [14] analyze the relations between a block’s texture characteristics and its best coding mode and developed an adaptive strategy for fast mode decision. Luo et al. [15] propose a fast intra CU size decision framework based on keypoint distribution conducted from the keypoint detection on GPU. Tian and Goto [16] analyze the texture complexity of LCU and its four sub-blocks to filter out unnecessary prediction units. In [17], Min and Cheung use global and local edge complexities in several directions to decide the partition of a CU. By analyzing edge types existing in each CU and its sub-CUs, the CU will be classified as partitioning CU, non-partitioning CU, or undetermined CU for each depth level.

### C. Neighboring blocks based methods

Many researchers employ the depth information of the neighboring blocks to predict the proper partitioning of the current CU because there exist strong correlations among the current, spatial and temporal adjacent blocks. For example, Tian and Goto [16] also utilize the prediction unit sizes of encoded neighboring blocks to skip small prediction unit candidates for the current block. Shen et al. [18–20] propose fast CU depth estimation and inter mode decision methods by utilizing the spatio-temporal correlations. To achieve better performance when encoding video with fast-motion scenes, Correa et al. [21] propose to estimate the best maximum coding tree depth based on both spatial and temporal correlations observed in the coding tree depths. Although these spatio-temporal based methods perform well when the adjacent blocks belong to the same object, around the boundary areas, it may decrease the coding efficiency since the neighboring CUs are more likely to be encoded with different sizes and prediction modes.

### D. RD cost based methods

RD cost based fast coding algorithms treat the CU splitting as a two-class classification problem. Most representatively, Kim et al. [22] utilize the minimum RD cost of inter- and intra-prediction at each CU to obtain the feature vector of the two-class classification problem. Then, these methods designed a classifier model to determine whether the current CU should be split or not based on the values of the cost function (1),

$$RDCost=SSE+\lambda •Bit_{mode}$$ (1)

where the *Bit*_*mode*_ is bits cost of the intra prediction mode, and the *SSE* is the associated distortion of luma and chroma.

### E. Machine learning based methods

Machine Learning, as a kind of data-driven method to solve classification problems or regression problems, is widely used in computer version and pattern recognition for decades. Many researchers have used machine learning in the field of CU partitioning due to its extraordinary performance. As the most popular machine learning method, Support Vector Machine (SVM) is utilized in plenty of works [23–30] to determine CU sizes. In [23], CU splitting is first modeled as a binary classification problem and SVM is applied to reduce the impact of outliers and maintain the RD performance. In [24], Zhu et al. propose to combine the off-line machine learning mode and the on-line machine learning mode for classifiers. In [25], Zhang et al. model the quad-tree CU depth decision as a three-level of hierarchical binary decision problem. In [26–30], various representative features are selected to achieve better RD performance. In addition [30], adjusts the decision threshold of SVMs to increase the rate-distortion performance.

Besides, decision tree (DT) is also a common method in CU size decision due to its simple structure and visual tree diagram. In [31, 32], Correa et al. use data mining to build a set of decision trees that terminating early the decision process by jointly utilizing the information of skip flags, merge flags, and RD costs of specific modes. To tackle the high computational complexity problem in H.266/VVC, Yang, Shen et al. [33] propose a novel fast QTMT partition decision framework to adopt the new intra coding process. Global texture information, local texture information, and context information are used in DT as a part of the proposed framework.

Some other machine learning tools including neural network [34], Markov Random Field [35], etc. are also introduced to block partitioning to achieve better video coding performance. However, machine learning based methods rely on the selected features. On the contrary, Convolution Neural Network (CNN) based fast algorithms do not need handcrafted features. Related works have been done in [36–38]. In [38], Xu et al. regard the block partitioning as a hierarchical CU partition map (HCPM) and build an early-terminated CNN architecture to predict the HCPM. Despite the advantages mentioned before, they take a heavy computational burden on the encoder due to their complex network.

In this paper, to achieve a better trade-off between the coding performance and the encoding complexity, we propose a decision tree based partition mode prediction and early termination scheme with an adjustable confidence threshold for intra prediction in VVC. The motivation and specific algorithm framework will be introduced in detail in the next two sections.

## Motivation and analysis

With the adoption of QTMT structure and more complicated intra modes, VVC has better coding performance for UHD videos. In the meanwhile, the more flexible CU size and deeper prediction depth lead to a huge increase in coding complexity. In order to design a more efficient coding scheme, it is necessary to make a detailed analysis of CU size decision process in VVC. On the other hand, to find the patterns among CU size, QTMT depth, and CU size decision process, several experiments are conducted.

In VVC, intra prediction and QTMT partition are performed sequentially at each CU depth. At first, CTUs are split into four equal-size square subCUs by QT structure. Then, each subCU can be recursively split into either smaller four square subCUs by QT or rectangle CUs by MT structure. Next, intra prediction and QTMT partition are performed sequentially at different CU depths, which is extremely time-consuming. Normally, some videos with low texture complexity and motion changes do not need to be divided too finely, and will not have a large QTMT depth. In order to well understand the CU partitions, we investigate the optimal CU size and QTMT depth distributions of Intra frames by employing seven typical test video sequences with different resolutions under different quantization parameters (*QPs* ∈ {22, 27, 32, 37}), including *FoodMarket4* from Class A1 with global motion, *DaylightRoad2* from Class A2 with translation motion, *BasketballDrive* from Class B with high motion, *PartyScene* from Class C with rich texture, *RaceHorses* from Class D with low resolution, *Johnny* from Class E with a stationary object, *ChinaSpeed* from Class F with a dark scene. Each sequence is coded with all-intra (AI) configuration under Common Test Conditions on VTM9.0.

For simplicity, we define *D*_*i*, *j*_ to represent the proportion of CUs with QT depth of *i* and MT depth of *j* to all CUs in one sequence. The QTMT depth distribution results are shown in Table 1. Some observations are summarized as follows.

**Table 1:** QTMT depth distribution for different sequences and QPs (%).

Sequence	QP	*D*_*1*,*0*_	Quadtree Depth 2				Quadtree Depth 3				Quadtree Depth 4		
			*D*_*2*,*0*_	*D*_*2*,*1*_	*D*_*2*,*2*_	*D*_*2*,*3*_	*D*_*3*,*0*_	*D*_*3*,*1*_	*D*_*3*,*2*_	*D*_*3*,*3*_	*D*_*4*,*0*_	*D*_*4*,*1*_	*D*_*4*,*2*_
FoodMarket 3840x2160	22	2.7	25.8	36.0	21.8	8.5	3.3	1.3	0.4	0.2	0.0	0.0	0.0
	27	6.7	44.8	29.8	11.7	2.7	3.1	1.0	0.2	0.0	0.0	0.0	0.0
	32	13.1	50.2	25.6	6.8	2.0	1.8	0.4	0.1	0.0	0.0	0.0	0.0
	37	25.4	47.6	20.9	3.5	0.8	1.5	0.3	0.1	0.0	0.0	0.0	0.0
DaylightRoad2 3840x2160	22	0.1	0.2	2.6	12.7	32.1	1.2	9.2	18.5	17.9	1.9	1.8	1.8
	27	0.9	3.3	9.0	16.3	33.0	1.9	8.6	13.2	10.0	1.9	1.3	0.6
	32	2.0	4.9	14.1	22.2	37.8	1.9	5.8	6.4	3.7	0.9	0.4	0.1
	37	3.6	9.4	23.5	27.4	29.5	1.2	2.8	2.1	0.4	0.1	0.0	0.0
BasketballDrive 1920x1080	22	0.1	0.9	6.4	24.6	35.3	1.3	5.7	10.2	7.4	4.1	2.5	1.6
	27	0.5	2.2	15.7	31.3	29.2	1.2	4.0	7.0	6.2	0.9	1.0	0.7
	32	1.4	5.3	23.2	29.3	25.0	1.3	3.4	5.1	4.5	0.6	0.5	0.3
	37	3.2	12.0	28.4	25.1	20.6	1.9	3.2	3.0	1.8	0.5	0.2	0.1
Johnny 1280x720	22	1.5	2.0	4.5	8.9	19.5	2.6	9.9	17.3	25.9	1.9	2.8	3.2
	27	2.0	2.4	6.0	11.0	22.1	2.9	10.3	15.4	19.8	2.7	2.7	2.6
	32	2.7	3.8	8.8	12.9	29.0	3.7	9.3	13.4	11.1	2.5	1.5	1.3
	37	4.0	6.8	13.9	18.8	31.9	2.8	7.3	7.7	4.7	1.1	0.7	0.2
ChinaSpeed 1024x768	22	0.2	1.0	3.2	5.1	8.5	1.8	7.4	16.6	32.6	2.6	7.5	13.5
	27	0.3	1.2	4.4	5.7	9.4	2.3	8.5	17.0	29.0	3.3	6.6	12.3
	32	0.4	2.2	5.8	7.2	12.5	2.5	9.7	16.3	25.8	2.7	5.2	9.7
	37	0.9	2.6	7.4	8.2	14.6	2.9	9.5	16.7	24.3	2.7	4.2	6.1
PartyScene 832x480	22	0.0	0.0	0.1	0.2	1.4	0.2	4.6	22.4	47.4	2.8	7.4	13.4
	27	0.0	0.0	0.1	0.4	2.1	0.3	6.0	22.7	44.0	3.6	8.1	12.7
	32	0.0	0.0	0.3	1.3	5.6	0.6	7.7	21.8	39.8	4.0	7.5	11.4
	37	0.0	0.1	0.6	3.9	13.2	1.3	9.8	21.3	32.4	4.7	6.0	6.7
RaceHorses 416x240	22	0.0	0.2	0.3	1.2	2.4	1.4	8.3	23.3	43.4	2.4	6.0	10.9
	27	0.0	0.3	0.5	1.4	3.9	1.7	9.0	22.0	38.8	4.0	6.8	11.8
	32	0.0	0.1	0.8	4.1	6.5	2.6	8.4	20.4	30.2	5.1	9.6	12.2
	37	0.0	0.2	2.9	6.6	19.6	1.8	12.2	16.5	26.3	4.6	3.8	5.6
**Average**	**-**	**2.5**	**8.1**	**11.2**	**12.6**	**17.4**	**2.0**	**6.6**	**12.3**	**17.6**	**2.1**	**3.1**	**4.5**
**Standard Deviation**	**-**	**4.9**	**13.7**	**10.3**	**9.2**	**11.6**	**0.9**	**3.2**	**7.6**	**15.1**	**1.5**	**3.0**	**5.0**

1. Generally, most of CTUs (about 87%) are coded in CU size with *D*_*2*, *j*_ and *D*_*3*, *j*_, *j* ∈{0, 1, 2, 3}. It can be observed that the percentage of *D*_*1*, *0*_ is the smallest with only 2.6%, and this number exceeds 25% only when the *QP* is very large in some specific sequence. Therefore, most of CU should be divided by QT twice and almost few CUs should be divided by QT three times.
2. Totally, 14.9% of regions are only coded with QT CUs and 85.1% of regions are coded with MT CUs after QT partition. It is noticeable that CUs tend to be split by MT to get better coding performance, which is why the complexity sharps compared with HEVC.
3. The standard deviation of most depth levels is small. Only when the MT depth gets larger, the standard deviation (STD) gets larger. Theoretically, the CU depth under All-intra configuration is mainly decided by texture contents. Therefore, those sequences with a large region of background are more likely to have lower depth, and the sequences with rich texture and fast motion tend to have bigger depth and smaller CU size.
4. Sequence resolution has a great influence on depth distribution. Sequences like *FoodMarket4* in Class A1, *DaylightRoad2* in Class A2 tend to be coded with smaller QTMT depth because that a CU in UHD video can carry more specific context while the same size CU in low resolution has to carry more context due to the limits of resolution, which also result in losing a lot of details.

**Fig 2:**
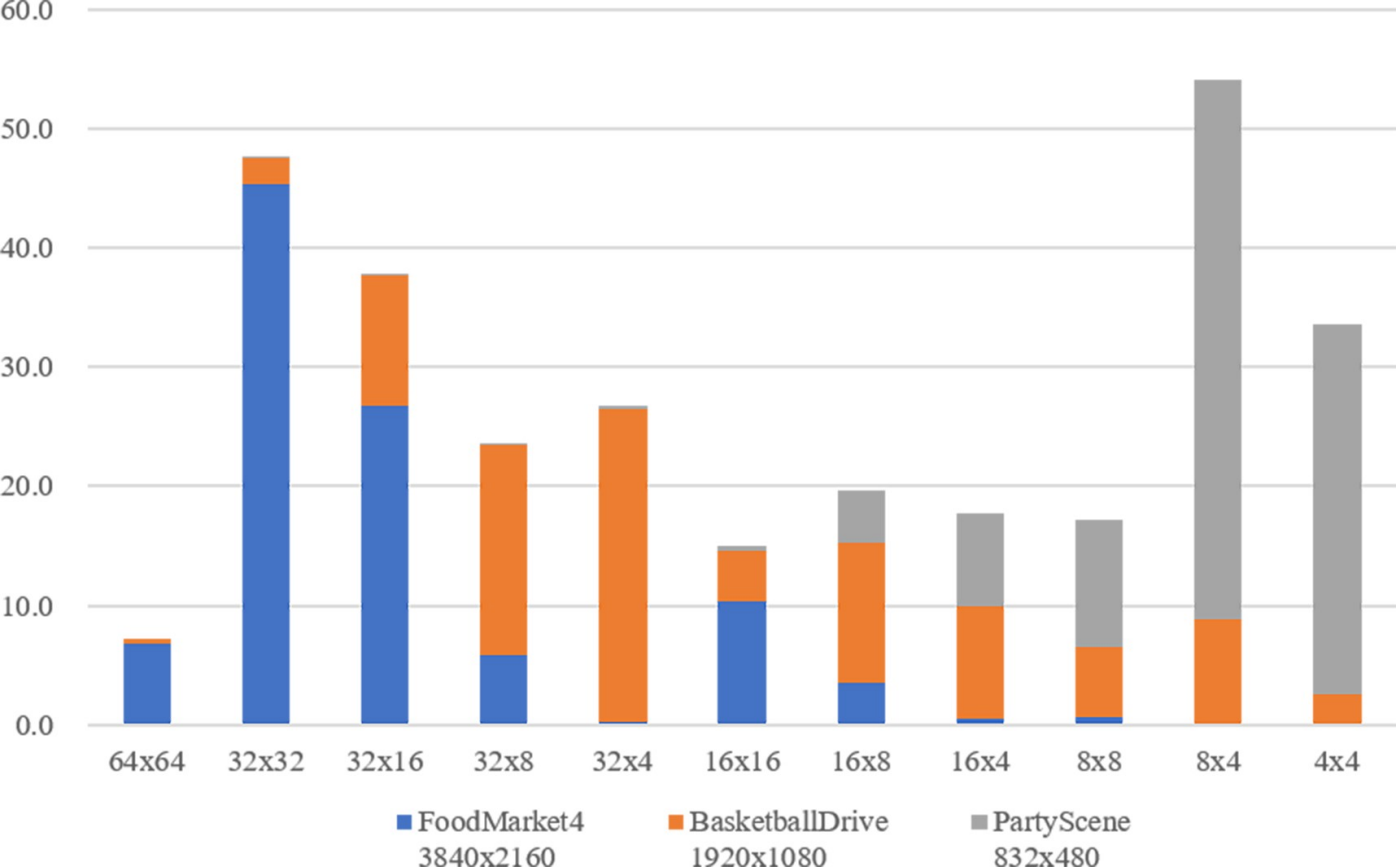
CU size distribution for different sequences with QP = 27.

In addition to CU-Depth analysis, similar results can be obtained by analyzing the CU size distribution from the final partition result of the above sequence. We choose three representative sequences with different resolutions to compare the proportion distribution of CUs of different sizes in their final partition results, as shown in Fig 2. When the resolution gets larger, VTM tends to use larger blocks to segment the image. As for low resolution video sequences, smaller blocks (corresponding to larger depths) tend to be used to achieve better coding performance. In MT partition, the probability of binary tree (BT) partition is much greater than that of ternary tree (TT) partition because the blocks corresponding to the ratio of width to height of 2:1 account for the vast majority of all blocks. Also, the blocks obtained from TT division generally use BT to get further partition (if it continues to be divided).

Based on the above analysis, if the CU can be determined in advance to be divided using QT structure, the remaining MT partition does not need to be checked anymore, which will save a lot of encoding time, especially for UHD sequence. Besides, if the rectangular blocks can remove the redundant MT candidate partition mode, it can also greatly reduce the encoding complexity of video sequences with different resolutions.

## The proposed decision tree based fast CU partition algorithm

**Fig 3:**
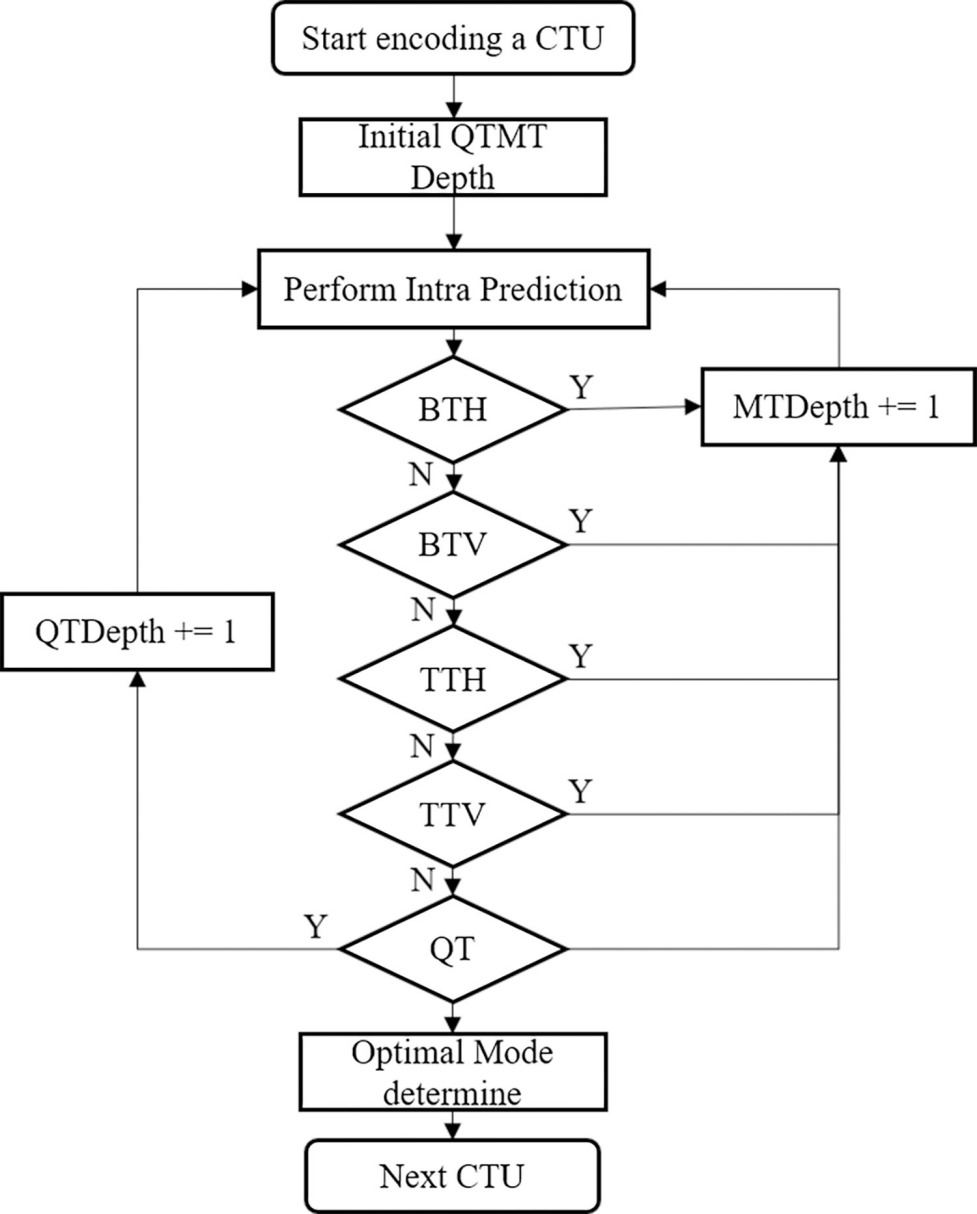
The flowchart of intra prediction in VVC.

In VTM, each CTU has to test a combination of all six partition modes and different prediction modes and then select the pattern with the smallest RD cost as the best coding structure. The specific process is shown in Fig 3. However, the RDO mechanism is very time-consuming. In order to reduce the complexity of this process, we try to predict the partition mode in advance or terminate the partition process to avoid checking redundant RDO. In the past, such fast algorithms using machine learning usually regarded CU partitioning as a multi-classification problem. However, due to the MT partition introduced in VTM, some fast algorithms in HEVC are unable to adapt to VVC or achieve good performance. We also regard CU partition as a multi-classification problem, but to achieve better results and fit the current VTM prediction framework, we develop a more comprehensive decision architecture based on previous researches, select decision tree as the classifier, and choose more representative features for offline training.

**Fig 4:**
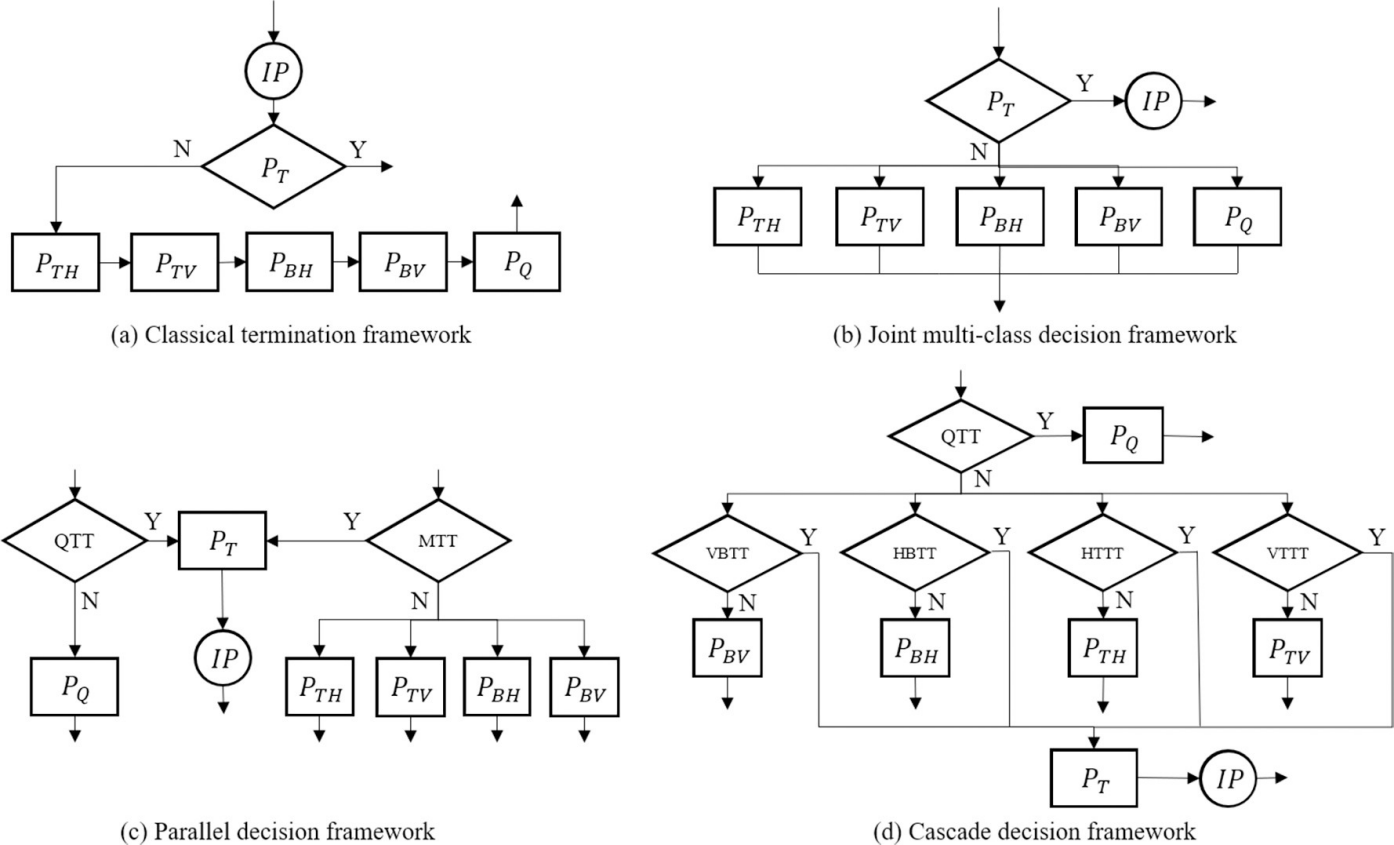
The existent CTU partition decision framework. (a) Classical termination framework, (b) Joint multi-class decision framework, (c) parallel decision framework, (d) cascade decision framework.

Currently, the proposed fast CTU partition decision framework can be summarized into the following four types, from Fig 4(A)–4(D). Fig 4(A) is a classic termination framework, in which intra prediction will be executed first at the current depth, and the information of intra prediction is used to determine whether the subsequent recursive partition should be terminated early. But the complexity reduction of this scheme is very limited because performing intra prediction is redundant if the current CU decides to be divided into sub-CUs. Later, the framework was improved to Fig 4(B) in [25]. However, the joint classifier was designed to select only one division mode before prediction, which resulted in a great loss of RD performance. After that [39], proposed a parallel decision framework as shown in Fig 4(C), trying to improve the accuracy of the partition decision. Still, the MT is a multi-classifier and only one MT mode is applied in the end. Recently, a cascaded decision framework in [33] was proposed, as shown in Fig 4(D). After the QT partition decision, four MT partition decision trees are used to determine the MT partition. Although this can achieve better accuracy and improve RD performance than before, it is possible that four MT decision trees in parallel all decide to divide current CU using their own partition mode. The paper does not give a detailed statement on how the encoder handles this situation.

**Fig 5:**
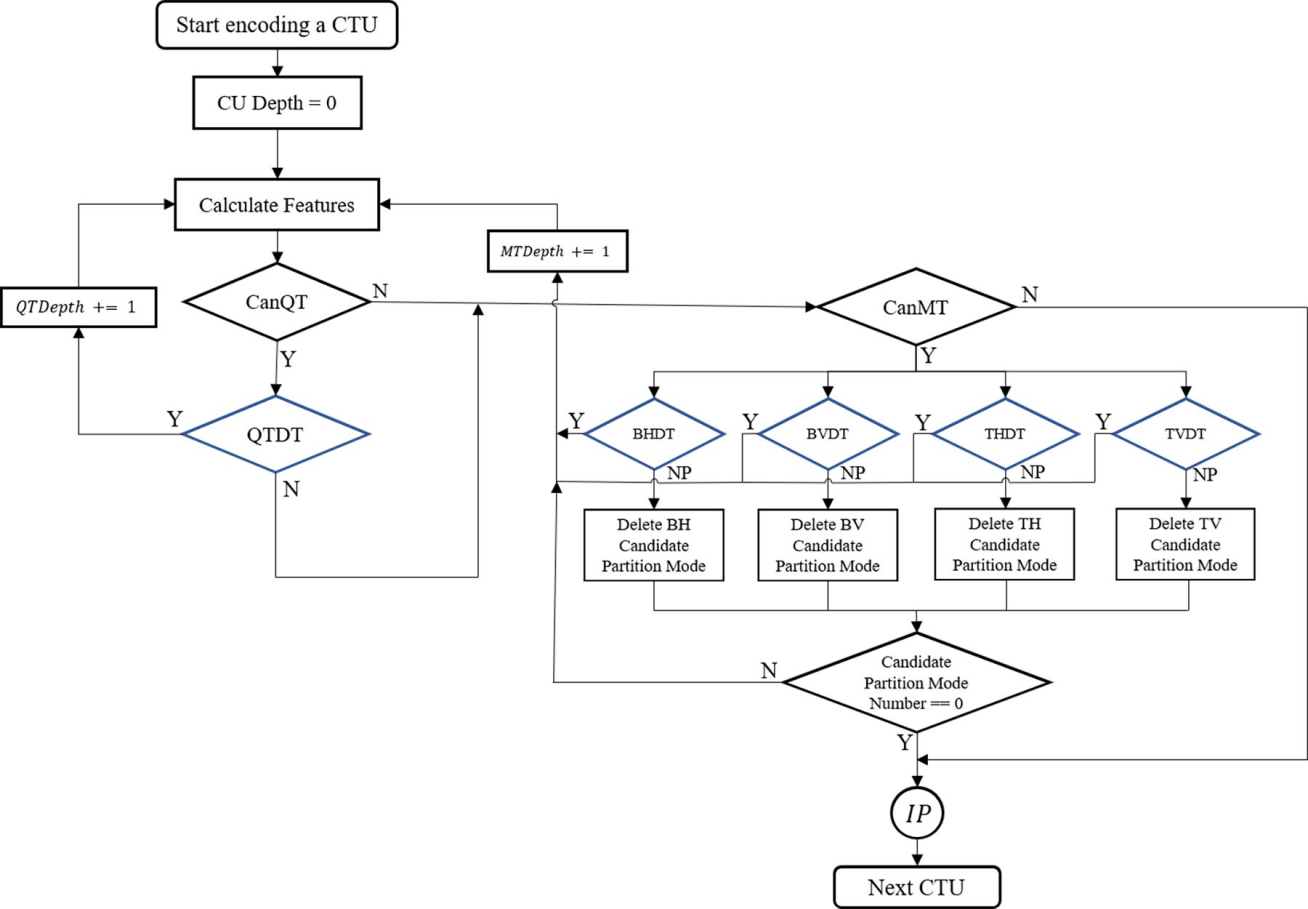
Proposed decision tree based partition mode prediction and early termination cascade decision framework.

In summary, based on the former research, we have supplemented and improved the fast CU partition decision framework as shown in Fig 5. First, we judge the feasibility of the QT partition in advance. If QT partition is allowed, we use QTDT to determine whether the current block chooses to be split by QT. There are two prediction results of QTDT. If the prediction result of QTDT is “Yes”, the subsequent MT partition is skipped, the *QTDepth* is increased by 1, and the recursive prediction is continued. Otherwise, the remaining part of our framework proceeds. Then, we also judge the feasibility of MT at the beginning. If the current block still cannot be split by MT, intra prediction will be performed. On the contrary, if MT partition is allowed, we use four MTDT, which are multiple binary classification trees, to screen out the redundant candidate partition modes in the VTM encoder for the corresponding MTDT decision results (NP). If the corresponding MTDT prediction result is “Yes”, the *MTDepth* is increased by 1 and the recursive prediction is continued. Since the QT partition cannot be performed after MT partition, the CanQT result after recursion must be “No” and only MT prediction will be performed. If all candidate partition modes are determined to be deleted, the intra prediction at current depth will be performed.

To make the trained decision tree have higher decision accuracy, we selected some representative features to fit the new QTMT structure better. In section IV, according to the statistical analysis of the depth distribution and the block size distribution, we found that the depth and size information of current CU, the partition mode of the parent block, and *QP* parameters have a great impact on the current CU block partition mode. At the same time, the texture feature and direction feature of the image itself has always been an important reference factor in the decision. On the other hand, for the current block, the complexity of its sub-blocks will directly affect whether the parent block is divided or not, but in order to adapt to the H.266 QTMT partition structure, the selection of sub-blocks also needs to be redesigned. Based on the above analysis, four types of features are finally used in the proposed algorithm, including global texture complexity, local sub-CUs texture complexity, current CU context information, and neighboring CU reference information.

### 1) Global texture complexity

The variance of the CU is widely used in machine learning due to its representation of the degree of energy dispersion between different pixels in a CU. However, the calculation of variance for each CU in the recursive process will also affect the complexity of the algorithm to a certain extent. Considering this, we adopt Sobel Operator to estimate the image gradient which could represent the global texture complexity. Unlike the repeated calculation of the variance in each CU, the calculation of the image gradient using the sobel operator can directly calculate the full-frame gradient of the frame when the encoder reads a frame of the image. When we try to calculate different CUs’ gradients, we just need to read the gradient calculation result of the corresponding region.

**Fig 6:**
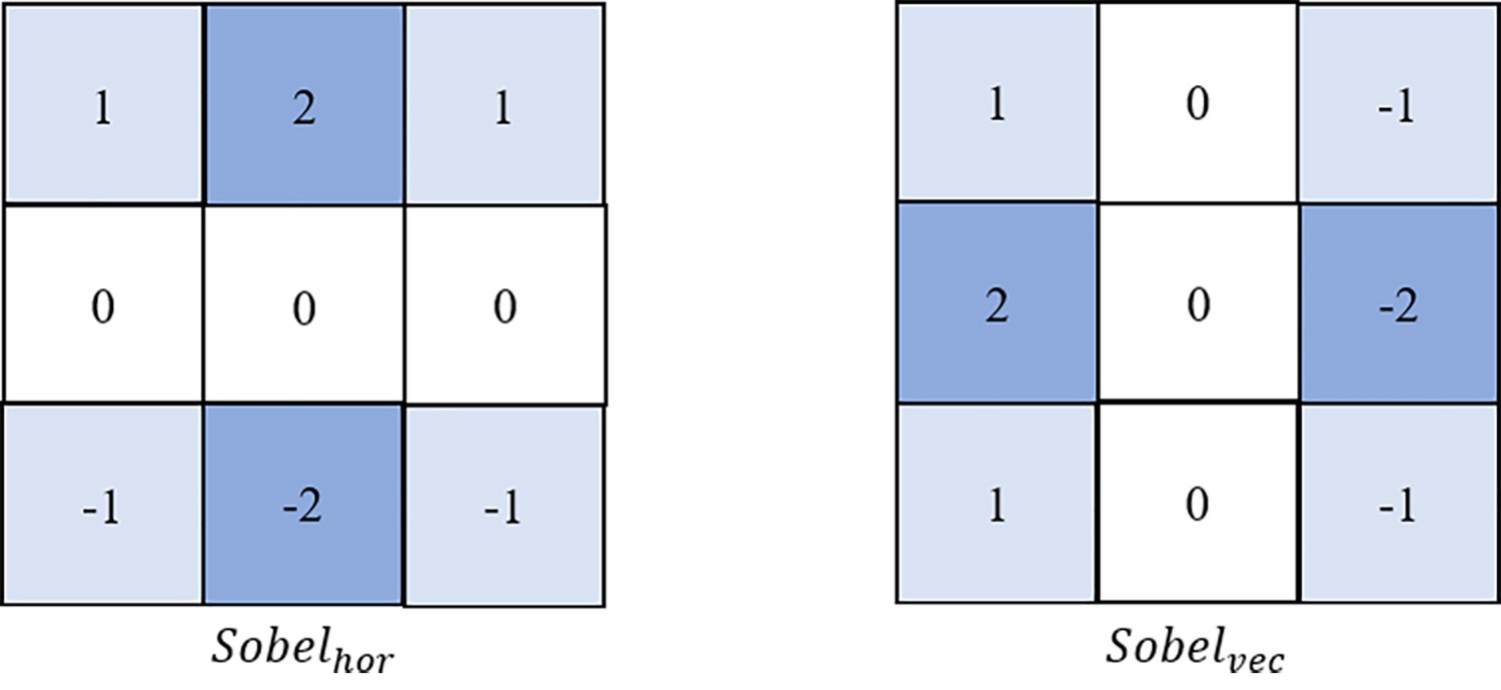
Two types of sobel operators for each pixel.

In this part, we use three features to measure texture complexity, including the normalized gradients (*xNG*), the average gradients in the horizontal direction (*xAGH*), and in the vertical direction (*xAGV*). The Sobel Operator has two directional modes: horizontal direction and vertical direction, as shown in Fig 6.

Using different sobel operator, we can calculate the horizontal gradient (*G*_*x*_) and vertical gradient (*G*_*y*_) by Eqs (2) and (3),

$$G_{x/y}(i,j)=Sobel_{hor/vec}*A$$ (2)

$$A=(\begin{array}{ccc} f(i-1,j-1) & f(i-1,j) & f(i-1,j+1)\\ f(i,j-1) & f(i,j) & f(i,j+1)\\ f(i+1,j-1) & f(i+1,j) & f(i+1,j+1) \end{array})$$ (3)

where *f(i*, *j)* denotes the luminance value of the pixel located at (*i*, *j*). *xNG* can be calculated as (4), where *N* is the pixel number of current CU.


$$xNG=\frac{\left|G_{x}|+|G_{y}\right|}{N}$$ (4)

### 2) Local sub-CUs texture complexity

**Fig 7:**
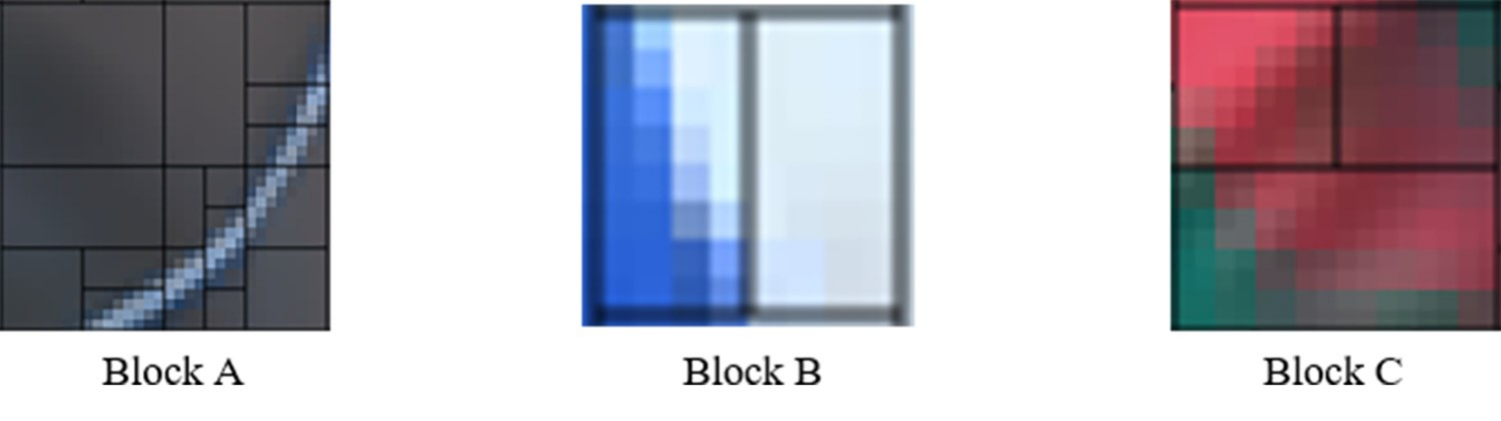
Final partition result of “*RaceHorses*” given by VTM9.0 under AI with QP = 32.

Besides global texture complexity, local sub-CUs texture complexity is also vital to MT partition decision. Fig 7 gives some partition results for “*RaceHorses*” sequence encoded by VTM9.0 with QP = 32, where Block A, a 32x32 CU, is split into four 16x16 CUs. Except for the upper left block, other blocks have relatively large texture complexity, so they are divided by MT again to adapt to their local textures. Similarly, Block B and C are further split by BV or BH for the same reason. From these scenarios, we could discover that when there is a large texture complexity difference between the sub-blocks of the current CU, the CU will be divided by corresponding partition type according to the different kind. To comply with the present QTMT structure, we design four features to measure sub-CUs texture complexity, including *xGDH*, *xGDV*, *xGDTH*, and *xGDTV*. *xGDH* and *xGDV* demonstrate the texture difference between left and right sub-CUs, and the difference between upper and bottom sub-CUs. *xGDTH* demonstrates that if the block is divided by TH, it is equal to the texture complexity of the upper and lower sub-CUs minus the texture complexity of the middle one. *xGDTV* is calculated by a similar rule only based on the hypothetical premise of TV partition.

### 3) Current CU context information

CU depth, size, and parent CU’s partition mode are closely related to block partition as mentioned in section III. Hence, we use *xQD*, *xMD*, and *xQTMTD* to indicate current CU’s depth information. *xQD* represents the quadtree depth of current CU, *xMD* represents the MT depth, and *xQTMTD* is defined as *xQTMTD = xQD + xMD*. On the other hand, we use *xMinWorH* and *xPSM* to represent current CU’s minimum width or height and its parent CU’s partition mode.

Moreover, we have already investigated the depth and size distribution under different *QP*. It can be noticed that *QP* plays an important role in VVC encoding process since this parameter directly influences the video coding bitrate and the partition of CU QTMT structure. Therefore, we use *xQStep* for training as well. There is a conversion relationship between *xQStep* and *QP* as (5), the *xQStep* will increase about 12.5% when the *QP* increases by 1, which allows *xQStep* to change in a larger scope than *QP*.


$$QStep\approx 2^{\frac{QP-4}{6}}$$ (5)

### 4) Neighboring CU reference information

Owing to the spatial correlations of video content, the partition structure of a CTU is always similar to that of spatial adjacent blocks. Therefore, we count the number of neighboring CUs whose *xQD* is larger than the current CU, denoted as *xNQD*. The location of neighboring CUs includes the left, the upper, the upper left, the upper right and the below left. Another number we use is the number of neighboring CUs whose *xQTMTD* is larger than current CU, denoted as *xNQTMT*. The larger the two numbers, the higher the probability of splitting the current CU. On the contrary, small *xNQD* and *xNQTMT* imply that the partition has large possibility to be terminated.

It is worth mentioning that all classifiers are trained offline, and the training samples are extracted from the “coding_tree” function in VTM9.0 encoding process. All sequences from A1 to F are used to extracted samples to make the models have better robustness. The samples are filtered according to the decision tree type so as to obtain a stable training effect. For example, when training QTDT, the extracted training samples meet the condition of *MtDepth = 0*. Morever, the training uses the CART classification function in the software Minitab 19, which can create an optimal decision tree for category responses with multiple categories or continuous predictors. The node splitting method selects the attribute splitting method based on the Gini index. In the training process, cross-validation is used to prevent over-fitting. The data set is divided into two groups, one is the training set, which accounts for 70% of the total samples, and the other is the validation set, which accounts for 30% of the total samples. In order to reduce the redundancy of the decision tree model, this paper adopts the post-pruning operation. Post-pruning removes some subtrees from bottom to top after the models have been trained. Compared with pre-pruning that may bring risks of underfitting, post-pruning can keep more branches.

At the same time, Minitab 19 can provide a visual tree diagram of the generated decision tree and the distribution of the positive and negative sample ratios of the corresponding nodes. For each leaf node of the decision tree, our algorithm will determine whether the proportion of positive samples reaches the manually set confidence threshold. If it is not reached, a complete RDO process will be performed on the corresponding mode to ensure the final RD performance. Specifically, the leaf node confidence thresholds QtTh and MtTh for QTDT and MTDT are both set to 90. Generally speaking, the smaller the value of QtTh and MtTh is set, the greater the RD performance loss, and the better the complexity reduction effect.

## Experimental results

To evaluate the performance of the models obtained by offline training and the proposed decision tree based fast CU partition algorithm for intra prediction in H.266/VVC, this section will show the experimental results obtained after integrating our algorithm into VTM9.0. The simulation environments are shown in Table 2.

**Table 2:** Simulation environments.

Items	Descriptions	
Hardware	Intel(R) Xeon(R) W-2123 CPU @ 3.60GHz, 16GB DDR3 RAM	
Software	Visual Studio 2015, VTM9.0	
	Minitab 19	
Video Sequences	Classes	A1, A2, B, C, D, E, F
	Resolutions	3840x2160, 1920x1080, 1280x720, 1024x768, 832x480, 416x240
Configuration	encoder_intra_vtm.cfg	
QPs	22, 27, 32, 37	
CTUSize	128	
MaxCUWidth/Height	64	

### 1) The prediction accuracy of the decision tree models

The accuracy of the decision tree models obtained by offline training through the above features is extremely important because it directly affects the final performance of the algorithm. The prediction accuracy represents the percentage of samples that are correctly predicted to the overall samples. Since cross-validation is selected when generating the decision tree models, the accuracy of several decision tree models in the training set and test set can be counted through the software Minitab 19. The final results are shown in Table 3. The TPR represents the true positive rate and equals the ratio of the number of positive samples that are correctly predicted to be positive to the actual number of positive samples. The FPR represents the false positive rate and equals to the ratio of the number of negative samples that are predicted to be positive to the actual number of negative samples. Similarly, the FNR represents the false negative rate and the TNR represents the true negative rate.

**Table 3:** The prediction accuracy of various decision tree models.

Model Type	Training set(%)				Test set(%)			
	TPR	FPR	FNR	TNR	TPR	FPR	FNR	TNR
QTDT	93.8	5.5	6.2	94.5	89.8	11.5	10.3	88.5
BHDT	85.3	7.9	14.7	92.1	84.2	9.8	15.8	90.2
BVDT	92.6	17.7	7.4	82.3	90.7	19.2	9.3	80.8
THDT	92.6	15.8	7.4	84.2	92.4	15.8	7.6	84.2
TVDT	94.8	14.9	5.2	85.1	93.2	15.6	6.8	84.4

**Fig 8:**
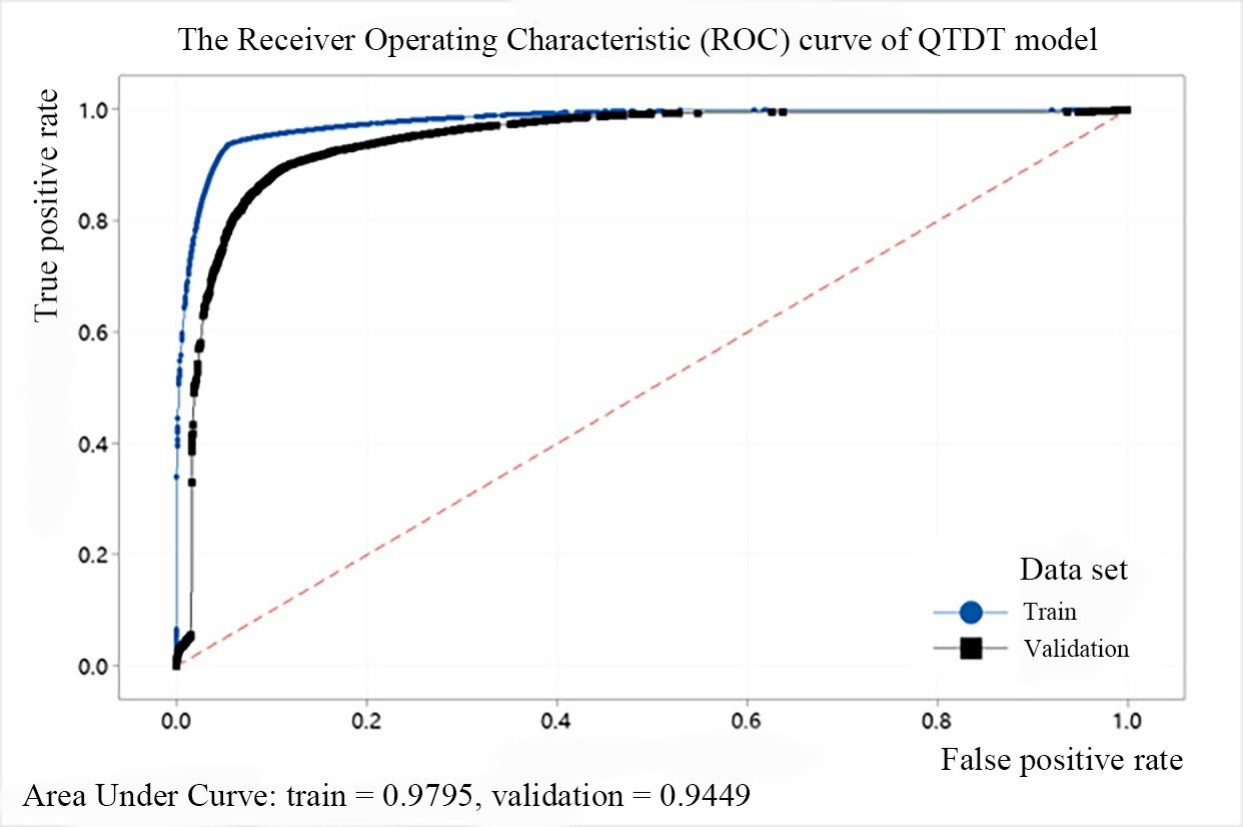
The ROC curve of QTDT model.

**Fig 9:**
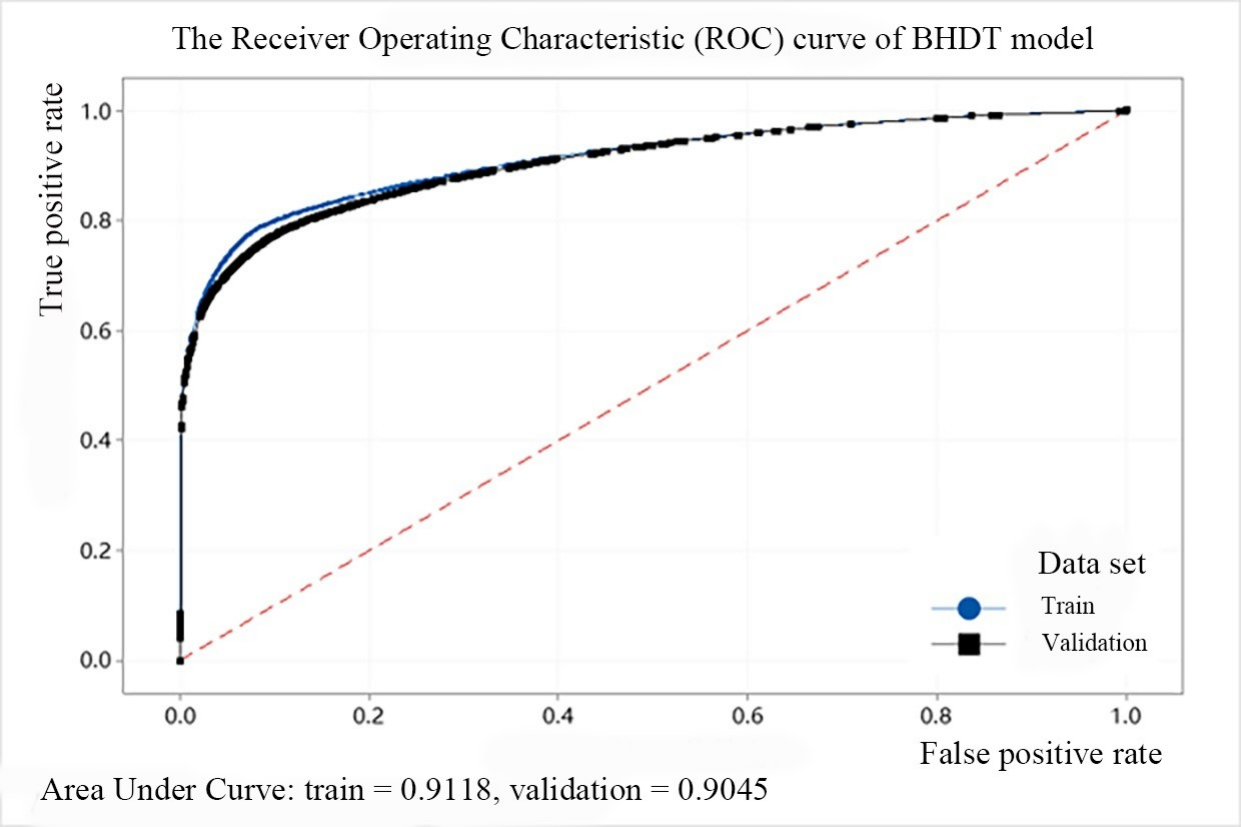
The ROC curve of BHDT model.

**Fig 10:**
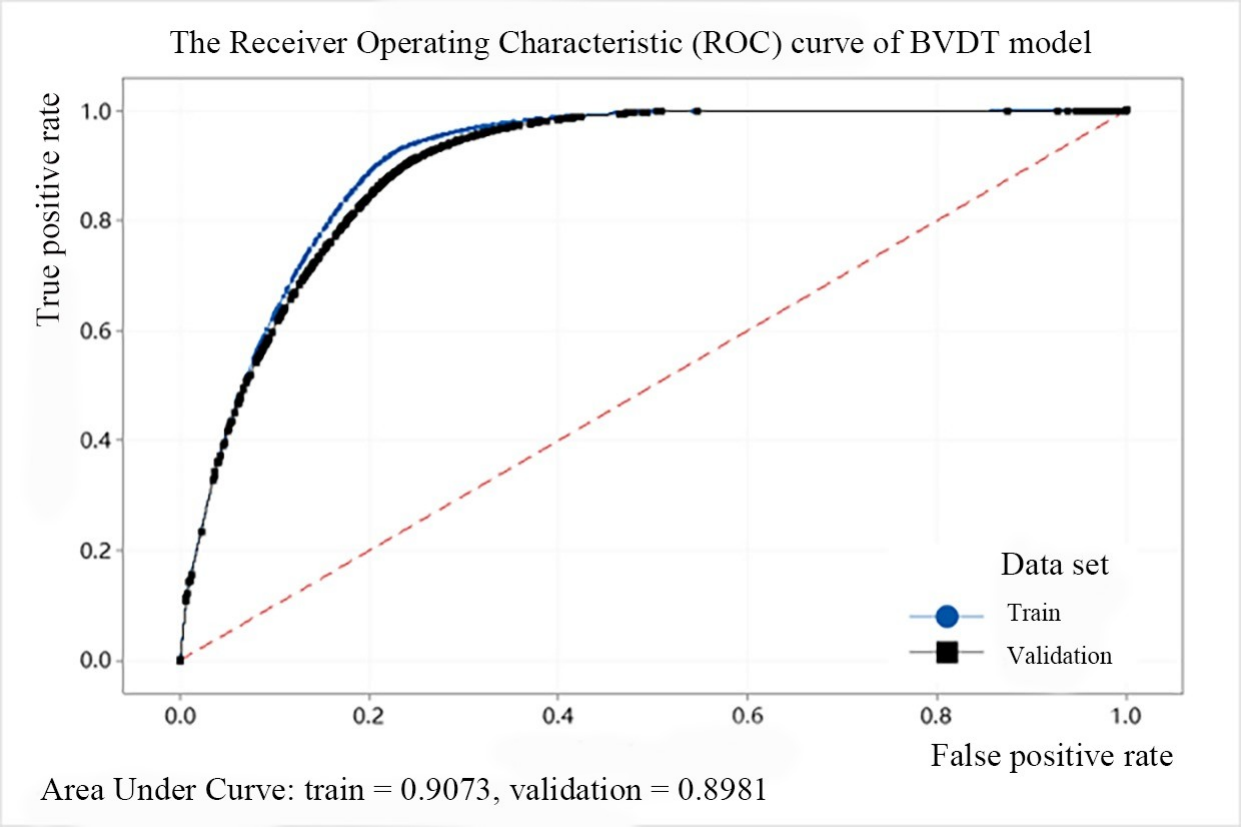
The ROC curve of BVDT model.

**Fig 11:**
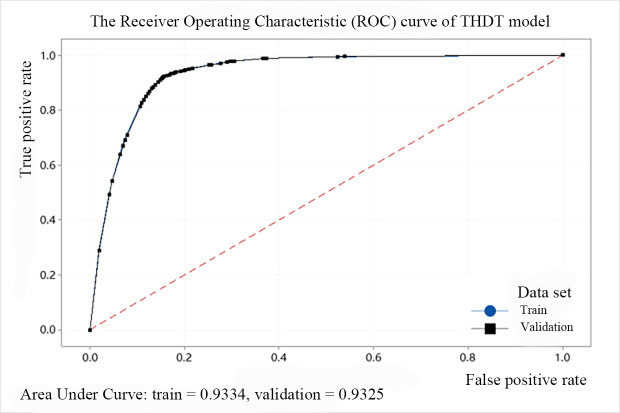
The ROC curve of THDT model.

**Fig 12:**
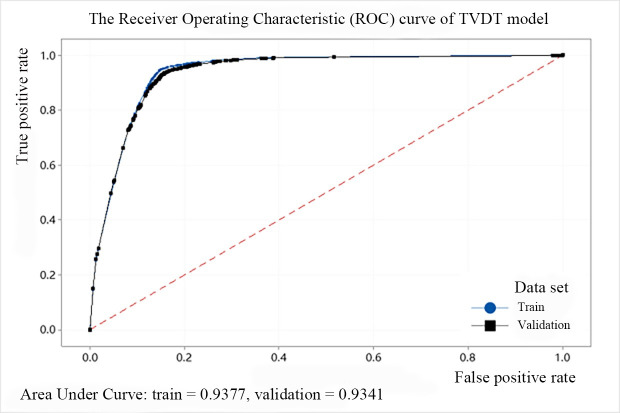
The ROC curve of TVDT model.

In addition, we use the ROC (Receiver Operating Characteristic) cure to describe the model effect of the decision tree. The abscissa of the curve is the FPR and the ordinate is the TPR. The area between the ROC curve and the abscissa is defined as AUC (Area Under Curve). When the area is larger, the AUC value is closer to 1, and the predicted point is closer to the coordinates (0, 1), indicating that the model performance is better. The ROC curves of the above five models are shown from Figs 8–12. It can be seen that the AUC value of QTDT reaches 0.9449, and the AUC value of four types of MTDT is also around 0.91, indicating that the accuracy of the decision tree models is reliable.

### 2) Performance evaluation of the proposed algorithm

We test a total of 26 video sequences from Class A1 to Class F for a more comprehensive evaluation of the proposed algorithm. Among them, Class A1 and A2 are newly introduced 10-bit depth UHD sequences, and Class B-F are the previous 8-bit depth HEVC sequences. The performance is measured by Bjontegaard Delta Bit Rate (BDBR) [40], Bjontegaard Delta PSNR (BDPSNR), and complexity reduction (CR) [33]. The BDBR represents the rate saving of the two methods under the same objective quality, and the BDPSNR shows the difference of PSNR-Y between the two methods at the same bitrate. CR is the average saving ratio of encoding time under different tested QPs, which is defined as (6),

$$CR(\%)=\frac{1}{4}\sum_{i\in Q}\frac{T_{r}(i)-T_{p}(i)}{T_{r}(i)}\times 100\%$$ (6)

where *T*_r_(*i*) and *T*_p_(*i*) represent the total encoding time of the reference VTM9.0 encoders and the proposed algorithm under *Q*_*i*_, respectively. *Q* contains four *QPs* (22, 27, 32, 37).

The experimental results of the proposed algorithm implemented in VTM9.0 are shown in Table 4. According to Table 4, our proposed algorithm gets an average complexity reduction of 50.49% with 0.08 dB BDPSNR decrease or 1.75% BDBR increase. At the same time, regardless of the video resolution and the type of video content, our algorithm has all achieved good results. The standard deviation (STD) of CR is only 4.90%, the STD of BDBR is only 0.63% and the STD of BDPSNR is only 0.05%, which illustrates the robustness of our method.

**Table 4:** Results of the proposed algorithm compared to VTM9.0 encoder.

Class	Sequence	Proposed		
		BDBR(%)	BDPSNR(dB)	CR(%)
A1	Campfire	1.56	-0.05	55.54
	FoodMarket4	1.87	-0.04	56.95
	Tango2	2.28	-0.04	62.13
A2	CatRobot1	2.25	-0.05	46.93
	DaylightRoad2	1.59	-0.03	56.10
	ParkRunning3	0.89	-0.04	47.90
B	BasketballDrive	1.14	-0.03	56.73
	BQTerrace	1.85	-0.09	56.39
	Cactus	1.39	-0.05	52.60
	Kimono	1.43	-0.05	52.60
	ParkScene	1.10	-0.05	54.56
C	BasketballDrill	3.04	-0.14	50.03
	BQMall	1.69	-0.09	45.02
	PartyScene	0.98	-0.07	46.20
	RaceHorsesC	0.91	-0.05	48.75
D	BasketballPass	1.39	-0.08	46.25
	BlowingBubbles	1.04	-0.06	42.24
	BQSquare	1.24	-0.10	46.96
	RaceHorses	1.04	-0.06	45.08
E	FourPeople	2.01	-0.10	47.40
	Johnny	2.38	-0.09	50.41
	KristenAndSara	2.33	-0.11	52.93
F	BasketballDrillText	2.92	-0.14	55.33
	ChinaSpeed	2.79	-0.22	49.65
	SlideEditing	2.04	-0.26	46.63
	SlideShow	2.32	-0.15	54.81
**Average**		**1.75**	**-0.08**	**51.15**
**Standard Deviation**		**0.63**	**0.05**	**4.90**

**Fig 13:**
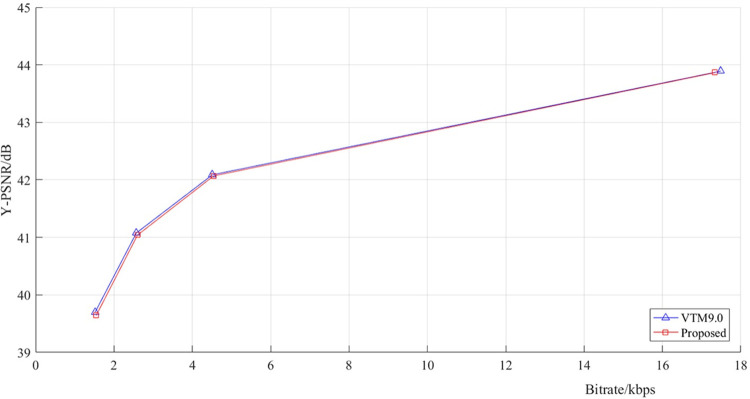
Performance of the proposed algorithm compared with VTM9.0 encoder of *Tango2*.

The RD curves of the proposed algorithm and the original test model VTM9.0 are shown in Fig 13, in which we choose the case “*Tango2*” in terms of the RD performance. It can be seen that the red curve and the blue curve almost overlap, indicating that our algorithm does not introduce large image distortion, but greatly reduce the coding complexity.

### 13) Performance comparison with the state-of-the-art works

Affected by the iteration of the version of VTM, other representative fast CU partition algorithms for intra prediction are based on lower VTM versions. Since our algorithm is developed based on VTM9.0, they cannot be directly compared. To this end, we close some of the coding tools in VTM9.0 through the configuration file and simulate the version environment as VTM2.0. Based on this version, we compare our algorithm with other state-of-the-art works, and the results obtained are more valuable. Table 5 shows the list of the coding tools closed in VTM9.0. Since the experiment is all-intra coding, the coding tools related to the inter prediction will not affect the experiment. Therefore, Table 5 does not list these tools.

**Table 5:** The closed coding tools in the configuration file when simulating the VTM2.0.

Coding tools	switches	value
Intra sub-partitions and its fast tool	ISP、ISPFast	0
Multiple reference line	MRL	0
Matrix weighted Intra Prediction and its fast tool	MIP、FastMIP	0
Low-frequency non-separable transform and its fast tool	LFNST、FastLFNST	0
Joint coding of chroma residuals	JointCbCr	0
Luma mapping with chroma scaling	LMCSEnable	0

The results of the most recent fast intra coding methods are presented in Table 6 for objective comparisons, including CSD-SL [33], CNN-CSD [36], and ETH-CNN [38]. Since ETH-CNN and CNN-CSD are proposed for HEVC with the sequences of 8 bit depth, Table 6 only presents the experimental results of Class B-F. Among them, ETH-CNN and CNN-CSD are the latest algorithms that use CNN to accelerate CU partitioning, and CSD-SL is the latest algorithm that uses similar ways to speed up CU division. The comparisons with these three algorithms have good reference value.

**Table 6:** Performance of the state-of-the-art algorithms and the proposed algorithm over simulated VTM2.0 environment.

Class	Sequence	CSD-SL [33]			ETH-CNN [38]			CNN-CSD [36]			Proposed		
		BDBR	BDPSNR	CR	BDBR	BDPSNR	CR	BDBR	BDPSNR	CR	BDBR	BDPSNR	CR
B	BasketballDrive	2.25	-0.06	64.01	1.11	-0.03	33.65	5.58	-0.13	34.28	1.80	-0.04	66.70
	BQTerrace	2.07	-0.11	56.07	2.08	-0.11	41.86	8.22	-0.45	52.51	2.03	-0.10	66.97
	Cactus	1.95	-0.07	56.66	1.47	-0.05	41.55	2.28	-0.08	46.10	1.48	-0.05	63.19
	Kimono	1.90	-0.07	63.87	0.98	-0.03	41.74	1.48	-0.05	18.48	1.56	-0.07	73.03
	ParkScene	1.33	-0.06	56.60	0.96	-0.04	42.39	2.30	-0.10	48.74	1.38	-0.06	63.40
C	BasketballDrill	2.01	-0.10	48.19	2.36	-0.11	41.47	2.93	-0.14	42.33	3.14	-0.15	62.66
	BQMall	2.15	-0.13	55.23	2.05	-0.12	45.31	3.37	-0.20	48.14	1.68	-0.12	60.79
	PartyScene	0.60	-0.05	45.73	0.93	-0.07	46.38	1.22	-0.09	47.49	1.07	-0.08	57.17
	RaceHorsesC	1.16	-0.08	48.39	0.82	-0.05	44.34	1.48	-0.10	51.69	1.17	-0.06	56.07
D	BasketballPass	2.33	-0.13	45.85	1.54	-0.09	43.79	2.68	-0.15	39.74	1.66	-0.09	55.09
	BlowingBubbles	0.77	-0.05	41.56	1.09	-0.07	43.07	1.15	-0.07	48.16	1.33	-0.07	52.94
	BQSquare	0.81	-0.07	46.06	1.51	-0.13	39.11	1.49	-0.13	42.86	1.41	-0.11	57.27
	RaceHorses	0.86	-0.06	43.17	1.01	-0.07	42.53	1.42	-0.10	50.85	1.15	-0.07	52.81
E	FourPeople	2.75	-0.15	57.64	1.98	-0.11	42.93	3.11	-0.17	51.09	2.27	-0.12	59.14
	Johnny	3.29	-0.13	58.98	1.63	-0.07	39.89	5.09	-0.56	49.17	2.58	-0.09	61.22
	KristenAndSara	2.51	-0.13	59.19	2.21	-0.11	40.56	3.11	-0.15	47.15	2.46	-0.12	59.79
F	BasketballDrillText	1.82	-0.09	47.66	2.25	-0.12	35.74	2.72	-0.14	42.61	3.02	-0.16	59.24
	ChinaSpeed	1.30	-0.11	52.67	9.22	-0.80	33.43	8.58	-0.77	40.02	2.95	-0.24	56.35
	SlideEditing	1.12	-0.18	48.91	10.69	-1.68	26.17	6.49	-1.02	36.14	2.05	-0.26	50.58
	SlideShow	2.61	-0.21	57.37	6.70	-0.54	41.03	7.37	-0.60	45.05	2.62	-0.18	59.45
**Average**		**1.78**	**-0.10**	**52.69**	**2.63**	**-0.22**	**40.35**	**3.60**	**-0.26**	**44.13**	**1.94**	**-0.11**	**59.69**
**STD**		**0.75**	**0.04**	**6.74**	**2.81**	**0.39**	**4.79**	**2.41**	**0.27**	**7.90**	**0.66**	**0.06**	**5.39**

It can be observed that CNN-based fast algorithms achieve acceptable results, which reduce the coding complexity by 40.35% and 44.13% on average, respectively. As for CSD-SL, its performance is better than the previous two algorithms, which has a complexity reduction of around 52% on average. The BDBR averagely increases by 1.78%, and the BDPSNR only decreases by 0.10dB. Compared to CSD-SL, ETH-CNN and CNN-CSD, the proposed algorithm has better coding complexity optimization performance. The CR almost reaches 60% and the STD is only 5.39%, which is smaller than that of CSD-SL. Besides, the proposed algorithm achieves a good balance between coding performance and complexity optimization. In general, the proposed fast CU partition algorithm outperforms state-of-the-art algorithms.

## Conclusion

In this paper, a decision tree accelerated with adjustable confidence threshold fast CU partition algorithm is proposed for VVC intra prediction. Firstly, we analyze the QTMT-based CU partition depth and CU size distribution and then navigate the intra prediction process. Based on the existing CTU cascade decision process, we redesign the framework and propose a DT based partition mode prediction and early termination cascade decision framework. More representative features are used to train QTDT and different types of MTDT. Finally, the whole framework is incorporated into VVC reference encoder (VTM9.0) to assist intra prediction. The experimental results show that the proposed algorithm can reduce about 51.15% computational complexity with only 1.75 RD performance loss compared to VTM9.0, which shows a good trade-off between coding efficiency and complexity reduction. Meanwhile, compared with state-of-the-art algorithms, our proposed algorithm has better complexity optimization performance based on the simulated VTM2.0 environment. It proves the effectiveness and superiority of our algorithm.

## Supporting information

S1 Fig Experimental data. The complete experimental data of our proposed algorithm.
(TIF)
