# Supplementary figures and images for: Decision tree accelerated CTU partition algorithm for intra prediction in versatile video coding
Source: PLoS One. 2021 Nov 8;16(11):e0258890. doi: 10.1371/journal.pone.0258890 (PMC8575300; doi:10.1371/journal.pone.0258890)

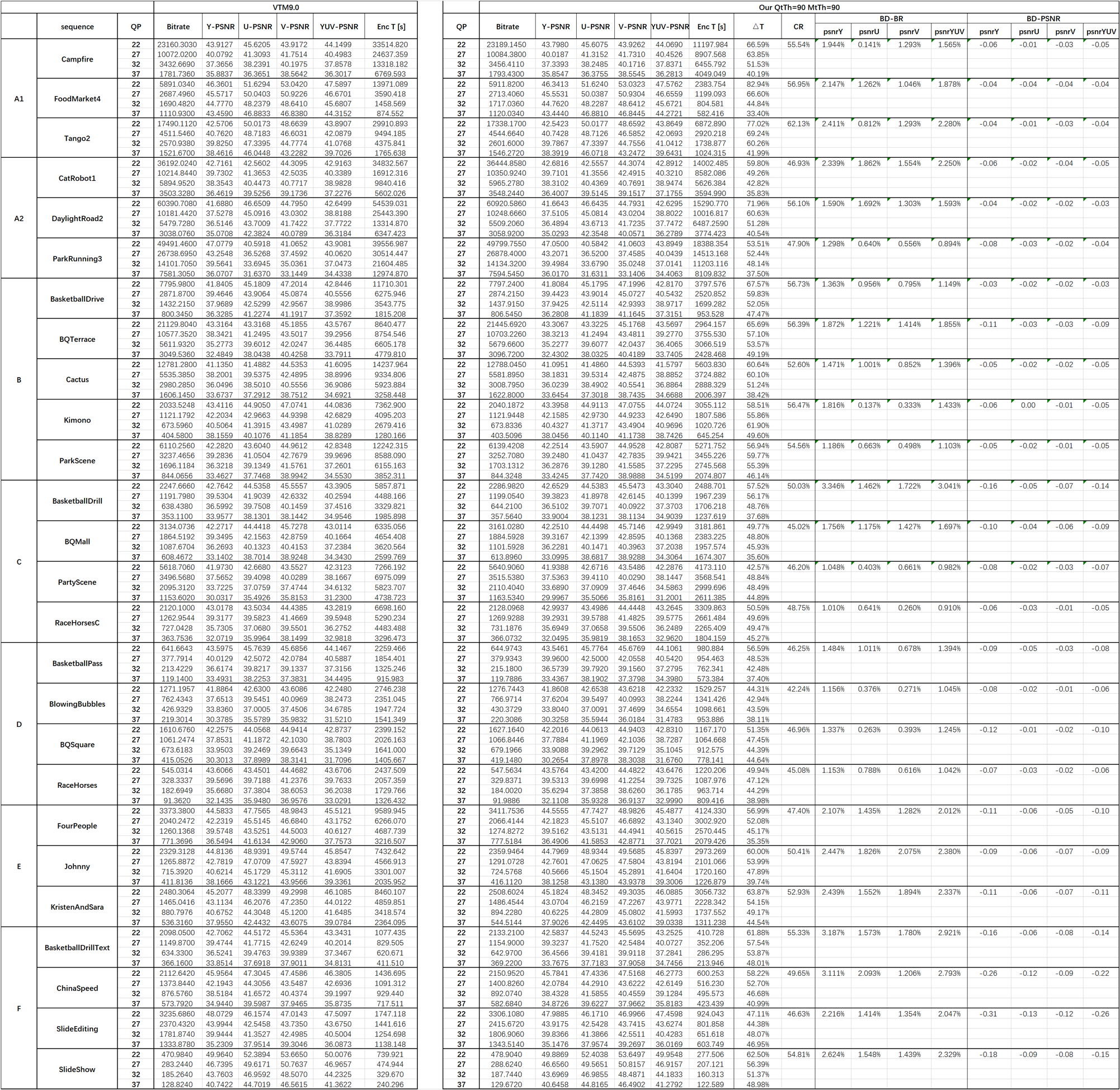

Supplement: S1 Fig — The complete experimental data of our proposed algorithm. (TIF) [file pone.0258890.s001.tif]
